# The big squeeze: a product content and labelling analysis of ready-to-use complementary infant food pouches in Australia

**DOI:** 10.1186/s12889-023-15492-3

**Published:** 2023-04-06

**Authors:** Kaitlyn A Brunacci, Libby Salmon, Jennifer McCann, Karleen Gribble, Catharine A.K. Fleming

**Affiliations:** 1 School of Health Sciences, Western Sydney University, Sydney, Australia; 2 Translational Health Research Institute, Western Sydney University, Western Sydney University, Sydney, Australia; 3 School of Regulation and Global Governance, The Australian National University, Canberra, Australia; 4 IPAN Deakin University, Geelong, Australia

**Keywords:** Complementary feeding, Infant Young Child Feeding, Infant food regulation

## Abstract

**Background:**

Encouraging the early development of healthy eating habits prevents diet-related chronic disease. It is well understood that highly processed foods with high amounts of sugars, salt and fats are a risk factor for non-communicable diseases. Commercial baby foods in ready-to-use squeeze pouches emerged in the global food market around 2012. The long-term effects of this now ubiquitous packaging on the quality of infant diets, baby food consumption and marketing are unknown. This study aimed to conduct a rigorous mixed-methods audit of squeeze pouches in Australia to inform product regulation and policy.

**Methods:**

Nutritional and marketing data were sourced from products available in Australian retailers. Analysis of nutritional content, texture and packaging labelling and serving size was conducted. Pouches were given a Nutrition Profile Index (NPI) score and compared with the Australian Infant Feeding Guidelines. Marketing text was thematically analysed and compared to existing infant nutrition policy around regulation of marketing claims.

**Results:**

276 products from 15 manufacturers were analysed, targeting infants from 4 + to 12 + months. Total sugar content ranged 0.8-17.5 g/100 g, 20% (n = 56) of products had added sugars, 17% (n = 46) had added fruit juice, 71% (n = 196) had added fruit puree. Saturated fat content ranged from 0.0 to 5.0 g/100 g, sodium 0.0-69 mg/100 g and dietary fibre 0.0-4.3 g/100 g. Only two products were nutritionally adequate according to a nutrient profiling tool. Marketing messages included ingredient premiumisation, nutrient absence claims, claims about infant development and health, good parenting, and convenience. Claims of ‘no added sugar’ were made for 59% of pouches, despite the addition of free sugars.

**Conclusions:**

Squeeze pouch products available in Australia are nutritionally poor, high in sugars, not fortified with iron, and there is a clear risk of harm tothe health of infant and young children if these products are fed regularly. The marketing messages and labelling on squeeze pouches are misleading and do not support WHO or Australian NHMRC recommendations for breastfeeding or appropriate introduction of complementary foods and labelling of products. There is an urgent need for improved regulation of product composition, serving sizes and labelling to protect infants and young children aged 0–36 months and better inform parents.

## Introduction

Encouraging the development of healthy eating habits early in life sets individuals on a path towards the prevention of diet-related chronic disease [1, 2]. The first 1000 days of a child’s life is a crucial period of early life development, wherein biology, environmental exposures and epigenetic effects that influence the risk of childhood obesity interact [3]. The World Health Organisation (WHO) recommends mothers exclusively breastfeed infants from birth to 2 years with ‘complementary feeding’(giving solid foods), in addition to breast milk, to begin around 6 months of age. [4]. The introduction of foods and feeding practices, known as the complementary feeding period (6–24 months), is a developmental window which establishes long-term dietary intake patterns for the child and lays the foundations for nutrition and feeding practices over the life course [5, 6].

During the complementary feeding period, foods need to be age appropriate, nutritious and safe and fed in an adequate amount in a responsive manner [5, 7]. Macronutrients and micronutrients are necessary for appropriate growth and cognitive development in infants, with iron, zinc, phosphorus, magnesium, calcium, and vitamin B6 being particularly important [8].

Consuming foods containing iron is particularly important, as the infant’s requirement for iron increases beyond what breastmilk alone can provide after 6 months of age [9]. To ensure a child meets their macro and micronutrients required for growth, UNICEF (2020) global nutrition guidance states that during the complementary feeding period children need to have a daily diet that is diverse, consisting of 5–8 food groups (breastmilk, grains, roots and tubers, legumes nuts and seeds, dairy, animal source proteins, vitamin A rich fruits & vegetables along with other fruits & vegetables). Along with this, UNICEF nutritional guidance recommends avoiding food or drink with low nutrient value and added sugars [7].

Similarly, the Australian Infant Feeding Guidelines [10] state ‘that consumption of nutrient-poor foods with high levels of fat/ saturated fat, sugar, and/or salt should be avoided or limited’ (pg 5) and no sugars should be added to food for children under 12 months of age [10]. Reasons for this avoidance include that frequent exposure to high sugar foods can have deleterious effects on the infant’s dental health and development of taste preferences as well as increase susceptibility to diet-related chronic disease in later life [11–13].

Methods of feeding complementary foods utilised by a parent or carer during the complementary feeding period influences a child’s feeding development. Oral motor skill acquisition during this time should include tongue lateralisation, chewing, gagging and swallowing with children being able to then apply these skills to different food textures [2]. Oral motor skills develop in connection with gross and fine motor skills to promote the development of self-feeding using fingers and hands (skills which mitigate future food fussiness during the toddler years) [5]. During this time the infant’s taste perceptions are also developing, providing a foundation of taste variety across the spectrum of sweet, bitter, salty and sour [14, 15]. The greater the taste exposure, the more likely a child will accept a diverse diet with bitter vegetables and sweet fruits, forming dietary behaviours that are protective against chronic illness [2, 6, 16].

The complementary feeding period can be challenging for parents and carers who may be unsure about their child’s needs and this constitutes an opportunity for commercial complementary food producers [17]. Squeeze pouches (also termed pouch-and-spout packaging or spout pouches) are a plastic retort baby food sachet or pouch with a mushroom-style cap containing pureed foods marketed as suitable for children aged 4 months-5 years [18–20]. Squeeze pouches enable children to consume wet ready-to-use food directly from the packet [21].

Manufacturers of squeeze pouches have targeted the complementary feeding period in Australia with their ‘convenience’ baby foods [18]. Squeeze pouches are the primary product sold in the Australian baby food market and have contributed to sales worth $1.2 billion (AUD)[21, 22]. The industry is dominated by five main companies, with the largest market shares held by PZ Cussons (Holdings) Pty Ltd, Heinz Wattie’s Pty Ltd and Bellamy’s Australia Limited [21].

The increased market share of squeeze pouch baby foods has been driven by changes in the labour market and ‘time-poor’ parents seeking what they perceive as the healthiest food for their child [21]. The marketing of these products as ‘convenient’ and ‘easy to feed’ helps parents make quick decisions about which products to purchase for their infant.

Nutritional composition analysis of squeeze pouch infant food has been undertaken in the US, Germany, Denmark, UK and New Zealand [19, 23–27]. These analyses had a striking common finding, with all pouch products considered high in both total and added or free sugars when compared to other infant and toddler foods [19, 23–27], with most of the total energy provided from free sugars[24]. Additionally, there was a predominance of apple, pear and sweet vegetable purees in squeeze pouches, and only small amounts of bitter vegetables and grains [25, 26]. Of concern is the finding that squeeze pouches available in New Zealand contained as little as 0.3 mg/100 g of iron, placing infants at risk of iron deficiency if complementary fed exclusively on commercial squeeze pouches [27].

In addition to the nutrient composition of squeeze pouches, there are concerns about the labelling and the marketing of these products to parents. In the USA, the Baby Food Facts Report (2016) found that most infant squeeze pouches do not support recommendations for encouraging healthy eating habits, and the marketing of the pouches is misleading about the true nutritional content of products, including the levels of sweetening [28]. Similarly, in the UK, a report by First Steps Nutrition (2018) found that many product names did not reflect their actual content, with 30% of 188 products analysed failing to mention the main ingredient (e.g. fruit puree) in the product name [26]. Despite the in-depth analyses of infant squeeze pouches products internationally, they have not been analysed within the Australian market. Rigorous analysis of Australian infant squeeze pouches, independent of other ‘infant and toddler foods,’ is particularly important given pouch products form the largest product range in the Australian baby food market [21]. In addition, some squeeze pouch products are marketed and viewed as a ‘whole’ meal for a child, encouraging parents to provide a large portion of their daily nutritional intake from a single product. Consequently, the nutritional composition and delivery of these products is paramount to a child’s nutrition and feeding development.

In Australia, regulation of commercial infant squeeze pouch products is limited. Nutritional content and nutritional content claims of squeeze pouches for sale in Australia have not been researched previously. The aim of this study was to conduct a rigorous mixed-methods product audit of commercial squeeze pouch products in Australia to inform regulation and policy.

## Methods

### Study design

We utilised a mixed-methods assessment of product labels to audit nutritional content and on-pack marketing claims. Quantitative and qualitive data was generated from front and back product labelling. Data was extracted for nutrient composition, marketing claims, recommended age of consumption, recommended serving size, serving mode (via spout or on a spoon), and texture.

### Data collection

Infant squeeze pouch products available between December 2018 and November 2019 were sourced from commercial retailers in Sydney, New South Wales, Australia. Retailers included Woolworths, Coles, Aldi, Big W, IGA, and Chemist Warehouse. These retailers hold approximately 85% of the Australian grocery retail market [29]. Products were photographed to capture the front, back, and side of package text. Internet searches were conducted to cross-check product availability and source products that may not have been available instore. Photographs of the products were entered into an Excel spreadsheet for analysis.

#### Inclusion and exclusion criteria

Squeeze pouch products included were pureed, semiliquid foods pre-filled in plastic pouches with a spout and screw cap [21, 24] that were marketed towards infants and toddlers. The recommended age was identified by labelling indicating that the product was suitable for children aged 4 + months, if the product was sold in the baby food aisle, or labelled with images suggesting it was appropriate for infants or toddlers aged 0–36 months, such as toddler cartoon characters (e.g. Peppa Pig™). Squeeze pouch products were excluded from data collection if they were aimed at older children and adults (e.g. packing indicated ‘added protein for muscle gain’) or products without marketing or instructions suggesting their use for infants or toddlers (e.g. squeeze pouch products containing preserved fruits found in the tinned fruit aisle).

### Data extraction

#### Nutritional content assessment

Nutrient composition was copied from the ingredients list and nutritional information panel (NIP) on packaging (expressed as g/100 g) by one researcher (KB). Where nutrient content was not reported by the manufacturer, the product was not excluded from analysis, and individual variables were labelled as missing. To ensure continuity and accuracy of data extracted, the entries were cross-checked and corroborated with information provided on manufacturers’ websites. In addition, the extracted data was intermittently independently reviewed by CF and LS to ensure data quality was upheld.

Nutrition information from each product’s NIP was recorded per 100 g for energy, protein, total fat, saturated fat, carbohydrates, sugars, dietary fibre, sodium (mg) and iron. The adequacy of macronutrients and micronutrients were ascertained using the Nutrient Reference Values recommended by the Australian National Health Medical Research Centre (NHMRC) for infants 0–12 months and children 1–3 years [8]. In addition to nutrient values, product content and labelling were compared to the Australian National Infant Feeding Guidelines in relation to compliance for age of introduction, texture and suitability of content e.g. recommendations regarding added sugars [10].

In Australia, currently there is no requirement regarding labelling of free sugar content in the nutrition label on packaged foods [30], neither is there a consistent or national definition of what constitutes ‘free sugars’. For the purpose of this study, we adopted the definition of free and ‘added sugars’ used by Public Health England: ‘free sugars includes all added sugars in any form; all sugars naturally present in fruit and vegetable juices, purées and pastes and similar products in which the structure has been broken down; all sugars in drinks (except for dairy-based drinks); and lactose and galactose added as ingredients. The sugars naturally present in milk and dairy products, fresh and most types of processed fruit and vegetables and in cereal grains, nuts and seeds are excluded from the definition’ [31]. To record and analyse the free sugars in each product we utilised the listed ingredients and observed whether the products were labelled as containing or not containing free sugars. Product total sugar content was determined, in accordance with the Australia New Zealand Food Standards Code (Schedule 4) definitions, with values generated from the NIP [32].

#### Product classifications

For analysis, products were grouped according to the manufacturer name e.g. Bellamy’s Organic and their primary ingredient food grouping, as listed on the back packaging. The food groupings were guided by the five core foods outlined in Australian Dietary Guidelines (ADG) such as fruit, vegetable, meat, dairy, grain [33]. Product labelling policy in Australia requires ingredients to be listed in order of greatest to least amount [32] and an assumption was made that the order in the ingredients list was accurate. In this paper, we use the language ‘main ingredient’ in reference to the first listed ingredient.

Products were also classified and grouped by the age recommendation stated on the product label. Age categories on product labels were presented as developmental feeding milestones of 4 + months, 6 + months, 8 + months and 12 + months.

#### Texture

Texture was recorded as smooth puree or with lumps according to the packaging description, or as directly visualised through packaging windows (when present).

#### The Nutrition Profile Index (NPI) score

The Nutrition Profile Index (NPI) was used to assess the quality of squeeze pouches [34–36]. The Nutrition Profile Index uses a scoring system where points are allocated for energy, saturated fat, total sugar and sodium, and subtracted for fruit, vegetables, nuts, fibre, and protein. Where specific nutrients were not reported in the nutrient information panel, points were not allocated to the product. For ease of interpretation, scores were adjusted to fit a 0-100 scale, where a score less than 74 denotes poor nutritional quality, a score between 74 and 82 moderate nutritional value, and greater than or equal to 84 is nutritionally adequate [28].

#### Age appropriateness of product

Age suitability was assessed independently using the CODEX international food standards that state ‘The label should indicate clearly from which age the product is recommended for use. This age shall not be less than six months for any product’ [37]. Labelling indicating that products were suitable for infants under 6 months were assessed as not age appropriate. In addition, products targeted at 6 + months were assessed on their product texture and if this was in accordance with the outlined recommendations in the Australian infant feeding guidelines of ‘from 6 months of age, infants should be offered purees and then mashed foods, progressing to minced and chopped foods by 8 months most infants can manage ‘finger foods’ by 12 months’ [10]. Product serving sizes were also recorded for each product, described in grams per serve, and servings per package.

#### Marketing claims

All on-pack information regarding the product health claims and any additional messaging were recorded. Product claims were compared to Standard 1.2.7 ‘Nutrition, health and related claims’ of the Australia New Zealand (ANZ) Food Standards Code [32]. The accuracy of claims and ingredients listed on the front label were compared to those recorded in the NIP to determine if front of pack information and claims breached the Food Standards Code. Claims were deemed ‘regulated’ if they complied with general health claims (e.g. contributes to general child development) or high level health claims (e.g. calcium for enhanced bone mineral density) [32], whilst ‘unregulated’ claims were those regarding taste, convenience, exclusion of elements such as preservatives, natural, organic or other messages relating to product promotion. Additionally, regulated and unregulated claims were thematically analysed from the perspective of how a parent might interpret the claims at the point of purchase. Text on front and back of packaging was utilised for the thematic analysis of all claims combined. For the purpose of this study, we have defined infants (0–12 months) and toddlers (13–36 months).

### Analysis

Statistical analysis of quantitative data was undertaken using Statistical Package for Social Sciences version 25 (SPSS, version 26 IBM corporation, NY). For nutrient content information, median range and distribution were calculated to determine the nutritional range of squeeze pouch products. Data was assessed for normality using the Sharpio-Wilk test. Depending on the distribution, continuous data are reported as mean ± standard deviation. Frequency and proportions were determined and compared for each product and age category.

Separate one-way analysis of variance (ANOVA) was used to identify relationships between nutrients (saturated fat, total sugars, sodium per 100 g), and the NPI score grouped in classifications of nutritional value (poor, moderate or adequate) for the target age of the squeeze pouch product (4, 6, 8 or 12 months).

Thematic analysis of qualitative data was utilised to find common themes that manufacturers used on the pouch products as information to consumers. Themes were grouped into sub-themes to further differentiate nuances of coded text for interpretation. Proportions were then calculated to determine frequency of use of the themes on packaging for different age categories.

## Results

### Product nutritional and textual composition analysis

Between December 2018 and November 2019, 276 commercial squeeze pouch products from 15 manufacturers were identified.

**Overall description of product composition by primary ingredient.**

43% (n = 119) of pouches were fruit-based, 32% (n = 88) dairy-based, 21% (n = 59) vegetable-based, 3% grain-based (n = 9), and one product’s primary ingredient was water.

Only two products were found to be nutritionally adequate according to the NPI scoring system, with 53% (n = 146) having poor nutrition (NPI score < 74) and 46% (n = 128) of moderate nutritional quality. Pouches with the lowest nutritional quality were dairy-based, with 97% (n = 66) of products scoring less than 74, followed by grains with an average NPI score of 71. Vegetable product groupings had the highest NPI score (77.9 ± 3.5) (Table 1).

Dairy-based squeeze pouches had the highest energy (366 ± 64 kj/100 g) and saturated fat content of all products (2.08 ± 1.12 g/100 g). Total sugar of all products ranged from 0.8 to 17.5 (g/100 g). Fruit-based pouches contained an average of 9.8 ± 3.1 g/100 g, and dairy-based pouches contained an average of 8.0 ± 3.3 g/100 g total sugar.

**Table 1:** Overall squeeze pouch product nutrient description by primary ingredient

	Average NPI Score (SD)	Poo r nutritional quality (NPI < 74) %	Moderate nutritional quality (NPI ≥ 74, < 84) %	Nutritionally adequate (NPI ≥ 84) %	Energy Kj/100 g (mean ± SD)	Total Sugar g/100 g (mean ± SD)	Protein g/100 g (mean ± SD)	Saturated Fat g/100 g (mean ± SD)	Sodium mg/100 g (mean ± SD)	Dietary Fibre g/100 g (mean ± SD)
**Vegetables**	74.4 ± 3.5	28.8	67.8	3.39	239 ± 48	2.8 ± 1.2	2.5 ± 0.9	0.45 ± 0.46	15.1 ± 9.3	1.7 ± 0.6
**Fruit**	74.4 ± 3.8	29.4	70.6	0	275 ± 55	9.7 ± 3.1	1.1 ± 1.0	0.22 ± 0.33	7.4 ± 6.8	1.7 ± 0.6
**Dairy**	66.0 ± 3.8	97.7	2.3	0	366 ± 64	8.0 ± 3.3	3.5 ± 0.9	2.08 ± 1.12	41.9 ± 14.3	1.1 ± 0.8
**Grains**	71.1 ± 2.5	77.8	22.2	0	246 ± 57	2.1 ± 2.5	2.0 ± 1.2	0.22 ± 0.26	10.9 ± 6.2	4.3

#### Description of products by target age group

A large proportion of squeeze pouch products available were targeted at infants aged 6 + months (40%, n = 110), followed by 4 + months (27%, n = 74), 8 + months (13%, n = 37) and 12 + months (4%, n = 12) (Table 2). A small proportion of pouches did not include a target age group despite packaging or product placement suggesting they were suitable for infants or toddlers (16% (n = 43). Of the pouches targeted at 12 + months, none had a vegetable as a primary ingredient (see Table 2). Squeeze pouches with the lowest nutritional quality were targeted at infants 12-months and older, with an average NPI of 67.8 ± 4.55. Squeeze pouches positioned for infants 4 + months of age had the highest NPI score, although the median score of 75 still only placed products in a moderate classification for nutritional adequacy.

Squeeze pouches across all age categories were energy dense, with 4 + month and 12 + months pouches containing between 248 ± 49 and 345 ± 65 kilojoules (kJ) per 100 g. Saturated fat was reported in 187 (67.8%) products, ranging from 0.0 to 5.0 Fat (g/100 g). The targeted age group with the highest amount of saturated fat was 12 + months, with a mean of 1.4 ± 0.94 g/100 g. Total sugar content in squeeze pouches for younger infants 4 + months was 8.7 ± 3.6 = g/100 g and in 12 + month squeeze pouches 8.4 ± 3.8 g/100 g. The saturated fat content was different between products for different age groups (p < .001) but not total sugar.

**Table 2:** Overall squeeze pouch product nutrient description by target age group

Product target age (n)	NPI Score (mean ± SD)	Main ingredient vegetable (% of age group) (mean ± SD)	Energy (kj) (mean ± SD)	Total Sugar (g/100 g) (mean ± SD)	Protein g/100 g (mean ± SD)	Saturated Fat g/100 g (mean ± SD)	Dietary Fibre g/100 g (mean ± SD)	Sodium mg/100 g (mean ± SD)
**4 + months (74)**	75.2 ± 3.50	12 (16.2)	248 ± 49	8.7 ± 3.6	0.87 ± 0.57	0.22 ± 0.34	1.6 ± 0.69	6.0 ± 6.1
**6 + months (110)**	71.6 ± 5.72	34 (30.9)	293 ± 77	6.4 ± 3.9	2.3 ± 1.3	1.3 ± 1.2	1.6 ± 0.49	19.8 ± 15.0
**8 + months (37)**	70.4 ± 4.67	13 (35.1)	289 ± 66	6.4 ± 4.9	2.1 ± 1.1	0.49 ± 0.50	2.1 ± 1.0	18.5 ± 15.7
**12 + months (12)**	67.8 ± 4.55	0 (0)	345 ± 65	8.4 ± 3.8	2.5 ± 1.2	1.4 ± 0.94	1.4 ± 0.4	23.8 ± 9.83
**Not stated (43)**	-	-	-	-	-	-	-	-

#### Micronutrients

Sodium content ranged from 0.0 to 69 mg/100 g and was 19.8 ± 15.0 and 23.8 ± 9.83 mg/100 g for 6 + month and 12 + month pouches, respectively. Sodium was significantly higher in dairy-based products (41.9 ± 14.3 mg/100 g) than vegetable-based products (15.7 ± 9.5 mg/100 g). Manufacturers with the highest average sodium content included The Collective Dairy (61.3 ± 2.4 mg/100 g), Parmalat (46.4 ± 15.4 mg/100 g), Brownes Food Operations (47.3 ± 1.0 mg/100 g), LD&D Australia (44.2 ± 1.2 mg/100 g), and Tamar Valley Dairy (34.6 ± 5.8 mg/100 g). All products were within the sodium guidelines of the ANZ Food Standards Code[32].

No products reported iron content, nor fortification with iron. Of the 68 (24.6%) products that reported calcium content, 65 were dairy-based products and 3 were fruit-based yoghurts. Only 16 meet the daily AI of calcium for the respective age group. The average reported calcium content per 100 g was 162 mg (SD 51.4 mg).

#### Free and added sugars

Overall, 72.8% (n = 201) of all products contained free sugars. Free sugars were found in 86.5% of squeeze pouches targeted at infants 4 + months, mostly in the form of fruit puree (73%) (Table 3). Additionally, 67% of squeeze pouches targeted at children 12 + months and 55.5% of squeeze pouches targeted at infants 6 + months contained added sugar in the form of fruit puree. Squeeze pouch products with no identified age suitability contained greater amounts of free sugars and contained both fruit juice and fruit puree (26%) and fruit puree concentrate (7%). Only 9.1% of all products included any bitter or green vegetables (spinach, broccoli) and, where included, these were mixed with free sugars. Products manufactured by Nestle, Only Organic, The Infant Food Co, Parmalat, Aldi, and Coles were especially high in free sugars (12.3 ± 1.1, 9.5 ± 4.8, 8.1 ± 4.4, 9.1 ± 2.5, 8.4 ± 4.2 and 9.2 ± 3.3, respectively). No products reported added monosaccharides or disaccharides in the ingredients list (including terms such as ‘sugar,’ ‘glucose’ and ‘sucrose’.

**Table 3:** Free sugar* content of squeeze pouch products by recommended age

			Types of sweeteners used in product composition			
**Product target age (n)**	**Total reported sugar content on label g/100 g (mean** ± SD)	**Total number of products by age group containing free sugars n (%)**	**Fruit Juice ONLY** **n (%)**	**Fruit Puree ONLY** **n (%)**	**Fruit Juice AND Fruit Puree** **n (%)**	**Fruit Puree Concentrate** **n (%)**
**4 + months (74)**	8.7 ± 3.6	64 (86.5)	0 (0)	54 (73.0)	10 (13.5)	0 (0)
**6 + months (110)**	6.4 ± 3.9	74 (67.3)	3 (2.72)	61 (55.5)	10 (9.09)	0 (0)
**8 + months (37)**	6.4 ± 4.9	23 (62.2)	1 (2.70)	14 (37.8)	8 (21.6)	0 (0)
**12 + months (12)**	8.4 ± 3.8	9 (75.0)	0 (0)	8 (66.7)	1 (8.33)	0 (0)
**Not stated (43)**	8.6 ± 3.0	31 (72.1)	1 (2.32)	16 (37.2)	11 (25.6)	3 (6.98)

*Free sugars includes all added sugars in any form; all sugars naturally present in fruit and vegetable juices, purées and pastes and similar products in which the structure has been broken down including all sugars in drinks (except for dairy-based drinks)

Age-Appropriateness of squeeze pouch products

An age recommendation was provided on 233 (84%) products, with an average of 6 months. However, 26.8% of products were marketed for infants aged 4 + months and consequently in breach of CODEX Standards, as well as encouraging feeding practices against WHO recommendations.

88.8% (n = 245) of products were categorised as smooth. While all products marketed for infants 6 + months of age were texturally appropriate, only 29.7% (n = 11) of products marketed for 8+-month-old infants were of an appropriate lumpy texture. All contents of 4+-month squeeze pouches were smooth purees without lumps. Only 55.4% (n = 153) of product labels recommended feeding the product with a bowl or spoon. Serving sizes were mostly 120 g (70.7%, n = 195)), irrespective of marketed age (Table 4).

**Table 4:** Age and texture appropriateness of products

Target age group (n)	Meets texture guidelines n (%)	Serving size (g) (mean ± SD)	Serving size 120 g n (%)
**4 months (74)**	0 (0)	118.8 ± 6.0	71 (95.9)
**6 months (110)**	110 (100)	114.6 ± 14.1	89 (80.9)
**8 months (37)**	11 (29.7)	111.7 ± 21.0	31 (83.8)
**12 + months (12)**	0 (0)	105.8 ± 35.5	4 (33.3)
**Not stated (43)**	NA	93.3 ± 29.4	0 (0)

### Product labelling and marketing analysis

#### Marketing themes

Analysis of packaging found six key marketing themes on front of pack labels: child development, child health, meal replacement, product premiumisation, convenience and good parenting. Product premiumisation promoted the inclusion of ingredients that were organic, natural, and good, for example “well-balanced, varied and nutritious” (Aldi). The front of pack text about general child development focussed on elements such as infant growth, such as “protein for growing bodies” (Parmalat), “vital part of your child’s early development” (Aldi), and “strong bones and teeth” (Parmalat). Front of pack labelling also targeted convenience “perfect size to travel with, as you explore the world together on the go” (Smiling Tums, Woolworths) and referred to being a ‘good parent’ or has messages seemingly to assuage guilt “Just as good as homemade” (Rafferty’s Garden, PZ Cussons). Other marketing messages included the absence of ingredients, for example “no added sugar,” “no added salt,” “no preservatives,” and “no artificial colours.” (see Tables 5 and 6).

**Table 5:** Marketing Themes and packaging examples

Themes	Sub themes	Front of Packaging example
**General Child Development**	**Growth**	vital part of your child’s early development (Mamia Organic, Aldi) Dairy goodness for growing kids (Pauls, Parmalat) Protein for growing bodies (Vaalia, Parmalat)
**Child health**	**Bone Health**	calcium for strong bones (Vaalia, Parmalat) strong bones and teeth (Vaalia, Parmalat) contributing to daily intake [calcium] for happy bones (Rafferty’s Garden, PZ Cussons)
	**Immune System**	Rich in vitamin C which helps to support the immune system and the absorption of iron. (Cerelac, Nestle)
	**Probiotics**	Contains inulin (Farex, Heinz Company) 3 probiotics (Vaalia, Parmalat) live probiotics for some tummy lovin’, Billions of live probiotics per pouch. (The Collective Dairy)
	**Other**	give your tummy some really good lovin’ (The Collective Dairy) Vitamin B12 for sustained energy and concentration (Vaalia, Parmalat Australia)
**Meal Replacement**	**Breakfast**	Ready to eat breakfast (Farex, Heinz) Brekky to go (Little Kids, Heinz) Yummy breakfast (Smiling Tums, Woolworths) Baby breakfast (Farex, Heinz)
	**Complete ‘meal’**	Yummy meal (Heinz) Perfect for lunch or dinner (Only Organic) Fruit and veggie meal (Smiling Tums, Woolworths)
**Premiumisation of product**	**Premium ingredients used**	made with full cream milk for dairy goodness (Heinz) purest 100% dairy (Brownes Dairy) goodness of New Zealand organic whole milk (Only Organic) well-balanced, varied and nutritious… wholesome premium products (Mamia, Aldi)
	**Expert development**	Exclusive, stringent quality (Mamia, Aldi) developed by our baby food EXPERTS (CUB, Coles)
	**High quality and trust**	only the best will do (Baby Macro, Woolworths) high quality ingredients (Bellamy’s Organic) ensure product quality (Rafferty’s Garden, PZ Cussons) loving developed… Products that you can trust (Mamia, Aldi)
	**Organic**	carefully selected organic ingredients (Heinz) Organic ingredients (multiple brands) Certified organic (multiple brands)
	**All ‘Natural’**	premium, natural, goodness of wholegrains (Rafferty’s Garden, PZ Cussons) Carefully selected natural fruit. (Cerelac, Nestle) vegan (Only Organic) no nasties (Bub’s Organic, The Infant Food Co) packed with goodness (Vaalia, Parmalat)
**Away from home convenience**	**Eating on the go**	perfect to take on your adventures together (Smiling Tums, Woolworths) perfect size to travel with, as you explore the world together on the go (Smiling Tums, Woolworths) enjoy custard wherever you are… home, work or on the go (Pauls, Parmalat)
	**School lunches**	freeze for lunchboxes (Yoplait, LD&D Australia)
**Good parenting**	**Wanting the best for your child**	We know that grown-ups want the best for their babies… (Rafferty’s Garden, PZ Cussons) Giving your little ones a pure start to life (Bellamy’s Organic)
	**Equivalent to ‘homemade’ products**	Just as good as homemade (Rafferty’s Garden, PZ Cussons) next best thing to homemade food. (Rafferty’s Garden, PZ Cussons)
	**Caring for your child**	protect and care for your little cub (CUB, Coles) helps your kids feel good on the inside (Vaalia, Parmalat)

When the themes and groupings were analysed by age, the most common were unregulated absence claims [32] for example, “no artificial colours, flavours or preservatives” and premiumisation claims based on use of organic ingredients. These claims appeared on all products targeted at age groups 4 + and 6 + months. Regulated messages [32] about ‘no added sugar and salt’ were also common on products for 4 + and 6 + months, with almost two thirds of these products claiming ‘no added sugar’ on the front of pack label. ANZ Food Standards Code Schedule 4 [32] regulated general and high-level health claims regarding child development were most common for products targeted at 6 + months, whilst claims relating to child bone health were more common for products targeted at 8 + months. Unregulated claims regarding ‘away from home convenience’ and ‘meal replacement’ were made predominantly for products that targeted infants aged 4 + and 6 + months.

**Table 6:** Marketing claims on products categorised by front of pack recommended age

Claims	Product front of pack recommended age (n = total number of products analysed in age grouping)						
	Age not reported (n = 43) n (%)	4 months (n = 74) n (%)	6 months (n = 110) n (%)	8 months (n = 37) n (%)	12 months (n = 12) n (%)	Total (n = 276) n (%)	
General Child Development							
General Development*	0 (0)	8 (11)	13 (12)	0 (0)	0 (0)	21 (8)	
**Child health**							
Digestive Health*	0 (0)	0 (0)	1 (1)	5 (14)	0 (0)	6 (2)	
Bone Health*	20 (47)	0 (0)	0 (0)	3 (8)	4 (67)	27 (10)	
Immune System*	0 (0)	3 (4)	1 (1)	0 (0)	0 (0)	4 (1)	
**Ingredient Premiumisation and Absence claims**							
**Premiumisation**	19 (44)	41 (55)	51 (46)	1 (3)	1 (8)	113 (41)	
**Mentions “Organic"**	4 (9)	39 (53)	55 (50)	9 (49)	3 (25)	110 (40)	
**Probiotic**	17 (40)	0 (0)	3 (3)	0 (0)	0 (v0)	20 (7)	
**Meal Replacement**							
**Breakfast**	0 (0)	2 (3)	6 (5)	2 (5)	6 (50)	16 (6)	
**Lunch/dinner**	0 (0)	0 (0)	0 (0)	1 (3)	0 (0)	1 (0.3)	
**Dessert**	0 (0)	0 (0)	0 (0)	1 (3)	0 (0)	1 (0.3)	
**Mentions “meal/mealtime”**	1 (2)	13 (18)	17 (15)	0 (0)	1 (8)	31 (11)	
**Convenience**							
**Away from home**	9 (21)	21 (28)	36 (33)	0 (0)	6 (50)	71 (26)	
**Good parenting**							
**Good parenting**	0 (0)	33 (45)	38 (14)	1 (3)	0 (0)	72 (26)	
**Additional absence claims**							
Claims “No Added Salt”*	0 (0)	51 (69)	53 (48)	17 (47)	0 (0)	121 (44)	
Claims ‘No Added Sugar’*	10 (23)	52 (70)	79 (72)	22 (59)	1 (8)	164 (59)	
**No artificial colours**	34 (79)	71 (96)	99 (90)	37 (100)	12 (100)	253 (92)	
**No artificial flavours**	29 (67)	74 (100)	110 (100)	37 (100)	12 (100)	262 (95)	
**No preservatives**	34 (79)	68 (92)	106 (96)	32 (86)	8 (75)	248 (90)	

*regulated claim e.g. “…helps to support the immune system” [32]

#### Label analysis and ‘mis-information’

There were discrepancies between the product title and actual listed ingredients of some products, with 9.4% of products marketed as containing vegetables only, 35.5% as fruits only, 0.7% as grain products only, and 21.4% as dairy-based products. The remainder were marketed as containing a combination of vegetables, fruits, meat, or grains. Despite 25 (9.1%) products reporting meat as the primary ingredient in the title, none listed meat as the main ingredient on the ingredients list and 22 of these (88%) listed the main ingredient as vegetables. Similarly, 44 (15.9%) products reported vegetables first in their title but only 32 (73%) of those listed a vegetable as the main ingredient. Only 92 (33.3%) squeeze pouch products contained a fruit as the first ingredient in the title, but 119 (43.1%) products listed fruit as the main ingredient.

Only 57 (21%) products were labelled as sweetened, yet 201 (73%) of products had free sugars, 17% (n = 46) in the form of added fruit juice, and 71% (n = 196) with added fruit puree. Several products contained more than one form of free sugar, as shown in Table 2. Some products (16.3%, n = 44) were labelled “no added sugar” or “no sweeteners” despite containing free sugar.

## Discussion

Squeeze pouch products form a large part of the Australian commercial complementary food market for young children aged 4 months to 5 years. Unfortunately, our findings were that most squeeze pouch products for infants and toddlers in the Australian market were inappropriate for use as complementary foods. Most products were nutritionally inadequate with a poor nutrient profile index scoring (only two were considered nutritionally adequate), and were micronutrient deficient (low in iron-rich ingredients and calcium) while being energy dense and high in (free/total) sugars. They were almost uniformly pureed and designed to be fed to children in a way that is developmentally inappropriate. Serving sizes were too large for infants while the products and commonly labelled as suitable for infants from 4 months, an age at which children should not be eating complementary foods at all. Finally, claims made on these products were commonly false or misleading. Each of these inadequacies has implications for child health and development.

### Iron and calcium deficient

No products reported iron content, nor fortification with iron, which is greatly concerning given the important role of iron for child growth and neurological development [38]. With iron stores depleted by 6 months of age, iron-rich foods are a crucial element to first foods at 6 months of age with the recommended daily intake for infants 7–12 months being 11 mg/day or the infant is at risk of iron deficiency (ID) [8]. ID is the most common micronutrient deficiency worldwide and young children are especially at risk due to their rapid growth [38]. Often ID is associated with lower-middle income countries where food insecurity and insufficient access to animal protein or iron-rich foods during infancy can results in immunosuppression, poorer cognitive function and stunting [38]. However, children in Australia and New Zealand who consume high amounts of low iron-rich complementary foods are also at risk of ID [22, 27, 38]. Additionally, calcium is fundamental for musculoskeletal development and growth [39]. Only 68 (24.6%) of products reported calcium content which was predominantly from the dairy-based product group, and of these only 16 met the daily AI for 7–12 months of 270 mg/day of calcium [8]. Thus, if young infants are fed predominantly squeeze pouch products as complementary foods, they are at risk of micronutrient deficiencies.

### Energy dense

Pouches across all age categories were energy dense, with 4 + month and 12 + month squeeze pouches containing between 248 and 345 kilojoules (kJ) per 100 g. A healthy active infant is recommended to have a daily kJ intake of between 2,500 and 3,500 kJ/per day depending on age [8]. Depending on serving size, a single squeeze pouch may contribute to excessive daily energy intake, if consumed frequently. Several elements contribute to the energy dense nature of pouch products, one being high levels of saturated fats followed by high levels of free sugars [26]. From the current audit, saturated fat was reported in 187 (67.8%) of pouch products, averaging 1.13 g/100 g (SD 1.13, range 0.0-5.0 g/100 g). The targeted age group with the highest amount of saturated fat was 12 + months, with an average of 1.4 g/100 g. With the Australian Infant feeding guidelines recommending ‘consumption of nutrient-poor foods with high levels of fat/ saturated fat, sugar, should be avoided’ for children 12 months and under [10], the finding that over half the squeeze pouch products in the Australian commercial baby food market, contain saturated fats and 72.8% contained added sugars should be considered a public health concern.

### High in sugars and sweetening

The research found that total sugar content in products across all ages groups was high. Total sugar was found to be highest in pouches for younger infants (4 + months) (8.7 ± 3.6 = g/100 g) and toddlers (12 + month) (8.4 ± 3.8 g/100 g), which is concerning given the Australian Infant Feeding Guidelines recommends children 12 months and under should ‘limit the intake of all foods with added sugars and not to add sugars to complementary foods’ [10]. Our findings concur with all other national [22, 40] and international (UK, NZ, Denmark, and US)[24–27] commercial infant squeeze pouch and baby/toddler food audits that found squeeze pouch products to be high in total and free sugars. In particular, Katiforis, Fleming [27] found squeeze pouch products to be higher in total sugars when compared to other commercial baby food products not delivered in squeeze pouches.

The current analysis found an alarmingly high proportion of products contained free sugars (72.5%) in the form of added sugar, fruit purees, and fruit juices. Most concerning was 86.5% of pouches for 4 + month-old infants contained free sugars, mostly in the form of fruit puree (73%), which could be impactful on long-term eating behaviours and metabolic outcomes [41]. Troublingly, squeeze pouch products that had no age specified, were using greater amounts of free sugars with products containing both fruit juice and fruit puree (26%) and fruit puree concentrate (7%).

Frequency and levels of sweeteners in squeeze pouch products are aided by the lack of a regulatory definition of ‘added’ or ‘free’ sugars in Australia [30]. The current ANZ Food Standards Code does not contain a definition of ‘added sugar’, although it does include criteria for making a claim regarding ‘no added sugars’ including honey, malt (extract), and concentrated fruit juices. Until a regulatory definition of ‘free or added’ sugars that encompasses ‘all sugars harmful to health’ is implemented, it is likely that squeeze pouches will commonly include sweetening agents such as fruit puree, placing young children at risk of long-term poor health outcomes.

Increased intake of sweet foods in infancy is known to contribute to a sweet taste profile preference [14]. In contrast, repeated exposure to savoury/bitter flavours increases their ongoing acceptance [6, 42]. With an evolutionary drive for young children to prefer calorie-dense sweet foods and reject bitter (or potentially toxic) foods [14], and an absence of regulatory oversight, it is not surprising that the food industry blend sweet fruit and vegetables in squeeze pouch products [22, 25, 43, 44]. Mixing dark green vegetables with sweeter vegetables or fruits or non-nutrient sweeteners derived from fruits (puree concentrate) increases product acceptance due to these evolutionary mechanisms [14, 25, 45].

Additionally, while our audit found 21.4% of all products contained a vegetable as the main ingredient, most were starch-based vegetables (pumpkin, potatoes, sweet potato) which have a relatively sweet flavour profile. Only 9.1% of products included any bitter or green vegetables (spinach, broccoli) and where included these were mixed with free sugars such as fruit puree, fruit puree concentrates and fruit juices. Only one product ‘eat your greens’ by Heinz had a standalone, non-mixed flavour profile of vegetables without any form of sweetening .

### Puree texture

For optimal feeding development, introduction of complementary foods should have an age-appropriate texture and consistency [5]. Despite this, only 43.8% of products met guidelines for appropriate texture for age. Given that 12-month-old infants should be consuming whole family foods with a variety of textures, all squeeze pouches marketed at this age group fail to meet the textural needs of these infants and compromise the child’s feeding development.

### Feeding method and portion size

The impact of energy dense products for infants during the early feeding development window can also be exacerbated with the spout and pouch packaging of squeeze pouches enabling the child to consume large amounts of food in an inappropriate manner in a short period of time [24]. The spout nozzle provides ease of consumption for the child without the need for oral processing such as chewing, or tongue lateralisation, along with the smooth texture of the pouch contents which can be easily squeezed at a rapid rate into the child’s mouth [27]. Only 50% of products in the current study contained advice to use a spoon or bowl. No product contained a warning not to squeeze contents directly into the infant’s mouth. According to consumer research in the UK, parents commonly allow infants to self-feed directly from the pouch or the parent squeezes food directly from pouch into the infant’s mouth [26, 31].

Most pouches in the current audit targeted at ages 4 + to 8 + months contained 120 or more grams per pouch product. In accordance to dietary guidance a child aged 4 months should not be consuming any complementary foods until 6 months of age [4, 10], therefore products that are available with a 120 g serve size are in excess of what a 4 and 6 month old child is required per meal serving [10]. If the child can easily consume food from the squeeze pouch as discussed above, concern arises surrounding the risk of excess energy intake. Moumin and colleagues (2020) found 20% of commercial squeeze pouch products in Australia categorised as dessert and breakfast products contained two serve sizes per package, rather than a single serve per package, enabling a child to consume two serving sizes of a higher energy dense product at one time. With overall meal size (kcal) shown to be associated with excessive weight gain in young children [46, 47] and the suggestion that large portion sizes contribute to childhood obesity[48], regulation of product serve sizes that are in line with infant feeding guidance are required to prevent the risk of long-term poor metabolic outcomes for children [24].

### Inappropriately labelled as suitable for infants from 4 months

Guidance from the WHO recommends that complementary food products be labelled to discourage their feeding to infants under 6 months. They state that ‘complementary foods should include information on not introducing complementary feeding before 6 months of age and not carry messages or contain information which may lead mothers and caregivers to believe that these products are suitable for infants below 6 months of age’ [49]. However, 26% of products in the study were labelled as suitable for infants from 4 months, potentially misleading parents to believe the products are suitable for children under 6 months (a ‘vital part of your child’s early development’ in one case) in contravention of international and national guidance. Labelling commercial baby food products as ‘4 months’ or ‘from 4 months’ has been shown to encourage parents to introduce complementary foods closer to four months than six months [43], displacing important nutrition from breastmilk. In addition to displacing breastmilk feeds, the early introduction of complementary foods risks the use of foods inappropriate for the infant’s developmental age. It must be questioned why the Australia and New Zealand Food Standard allows complementary foods to be labelled as appropriate from 4 months of age when the Australian infant feeding guidelines are that they not be introduced until around 6 months.

### Misleading claims on packaging

This study identified that parents are exposed to multiple marketing claims on labelling when considering purchasing squeeze pouch products, with all products included in the audit containing at least one marketing claim. Market messages on front of packet labels included ingredient premiumisation ‘organic, natural, good ingredients, well-balanced, varied and nutritious” and product absence messages such as “no added sugar,”” no added salt,” “no preservatives,” and “no artificial colours.” Such messages mislead parents by fostering the impression that the product is ‘better for you’ than the actual nutritional composition reflects [50, 51]. A review completed by Public Health England [31] found parents perceived products as healthy when front of packet labelling used wording such as ‘organic’ or ‘free from sugar.’

Analysis conducted by Simmonds, Brownbill [50] in Australia found squeeze pouch packaging contained multiple claims on the one packet. Of the multiple claims included, some were regulated and some unregulated - this included the claim of ‘*no added sugar*’. Although regulation does apply to the use of this specific claim in Australia, under the current definition in the ANZ Food Standards Code Schedule 4 [32] products can still contain high amounts of sweetening through use of free sugars, fruit juice concentrate and puree in formulation and use the ‘no added sugar’ claim, confusing parents on the true content of the product [51].

Parents are further misinformed when front of package labelling does not match the product contents and back of package labelling. For example, we found that despite 25 products reporting meat as the primary ingredient in the title, none listed meat as the main ingredient (particularly concerning given the low iron content of products, as discussed earlier). Similarly, 44 products reported vegetables first in their title but only 32 listed a vegetable as the main ingredient. Thus, in many instances product labels foster the perception that products are rich in iron or fibre from animal source foods or vegetables when this is not the case. When assessing the true free sugar content of products, we found only 21% of products labelled as ‘sweetened’ but that 72% of products contained free sugars. These products were often labelled as ‘no added sugar’ despite the high levels of sugars harmful to health in the product, directly misleading parents and carers. However, these nutrient absence claims are a violation of World Health Assembly (WHA) resolutions on ending the inappropriate promotion of foods for infants and young- children, which Australia, as a WHA member, is obliged to implement [49].

Despite complementary foods being intended to supplement breastfeeding, particularly for infants, none of the products we audited contained product messages that promoted breastfeeding and many squeeze products [31] had labels that described the product as a ‘whole’ meal or meal replacement. This may result in the displacement of breastfeeding if parents believe that the child’s nutritional needs are met adequately through the product. The WHO recommends that foods complement the intake of breastmilk up to at least two years of age and that ‘messages about complementary foods always include easily understood and clearly visible information on the importance of continued breastfeeding for up to two years or beyond’ [49] but such messages are absent in the Australian squeeze pouch market.

## Strengths and limitations

Strengths of our study include the large sample size of commercial infant and toddler squeeze pouch products. The study has provided a comprehensive collection of nutritional and marketing data for these products that was previously unknown. A limitation of the study is the cross-sectional, single time point study design. All product information was collected from December 2018 and November 2019, product reformulation or additional products entering the market after this time point are not included in the analysis. To overcome this limitation ongoing monitoring of commercial pouch and spout products is essential to assessment of the nutritional content and potential impact for infants and toddler feeding development. Additionally, any data that may have been missing from the product nutrient information panels (for example, fibre), were not able to be included in the calculation on the NPI score.

## Conclusion

The current audit demonstrates that commercial infant squeeze spout and pouch products available in Australia are nutritionally poor, high in sugars harmful to a child’s health, low in iron, not supportive of healthy development of infant feeding behaviours and labelling of products is misleading for parents. The long-term impact on a child’s eating patterns and food acceptance of squeeze pouch products containing sweeteners such as fruit puree that are an inappropriate texture for their age and stage of development is unknown. Further the link between food refusal/fussy eating behaviours and the repeated exposure of pouch products with a sweet taste profile/puree texture is unknown [15, 52]. However, there is a clear risk that if infants are regularly fed these products their health will be harmed. Squeeze pouch products are likely to lead to premature cessation of exclusive breastfeeding if introduced before 6 months of age, resulting in increased vulnerability to infectious disease. In addition, delayed introduction to older infants of food with lumps and ‘finger foods’ is associated with poor oral motor development, affecting eating and speech. Moreover, high intake of sweet flavour profiles promotes ongoing acceptance of foods rich in free sugars, contributing to diet-related chronic disease extending into late childhood and adulthood. Furthermore, the marketing messages on squeeze pouches are not aligned with recommendations for breastfeeding or appropriate introduction of complementary foods, and appeal to parents through ingredient premiumisation and the promise of convenience at mealtimes. Ready-to-use complementary squeeze pouches are an appealing product for parents in terms of convenience, but parents are potentially unaware of the true risk these products pose for their child’s health. A decade on from the introduction of squeeze pouch products, regulatory frameworks have not responded to established evidence on health and feeding impacts for children or kept pace with possible development opportunities in packaging that better support optimal feeding for infants and young children. As the market for commercial infant and toddler foods grows and squeeze products increase there is an urgent need for policy and regulation surrounding product composition, serving size and labelling to better inform parents. To promote the establishment of healthy infant eating patterns and protect the health of children, greater accuracy and accountability is needed in labelling of products marketed for toddlers and children under 12 months of age and improved composition of products is essential during this key period of growth and development.

## Recommendations

Given the growing use of complementary squeeze pouches in Australia for infants and children, there is a need to investigate further the frequency and pattern of use of squeeze pouches and their association with health outcomes. In addition, from a public health perspective to ensure all children can achieve optimal long-term dietary intake and health outcomes the following recommendations are made for the uptake by industry stakeholders and food regulators within Australia and across the globe:

1. Labelling must accurately represent the product’s primary ingredients, so parents are not misled at the point of purchase.
2. National food standards need clear definitions of ‘added’ and ‘free’ sugars that includes all sugars ‘harmful to health’. An upper total threshold limit for all forms of sugars ‘harmful to health’ needs to be set for commercial infant and young child food products. Free sugar labelling is currently under review by FSANZ [53].
3. Products should not be labelled or marketed for use by infants under 6 months of age, and need to comply with the WHA resolution on ending inappropriate promotion of foods for infants and younger children.
4. Foods in squeeze pouches with a spout for an infant or child older than 7 months need to have textures other than puree, in line with empirical evidence surrounding texture variety for optimal oral motor development. In the absence of a change in packaging, the products need a warning on the label on the front of the pack, stating that the method of feeding via the spout does not support normal infant feeding development and products should be used in this way in a limited manner.
5. Serving size should be standardised and labels should provide guidance for parents on age-appropriate servings.
6. Product reformulation is needed to include iron fortification and a varied flavour profile, with reduced use of concentrated sweetening to ensure children can start their life long nutritional journey with adequate nutrition and a variety of appropriate flavours.

## Data Availability

The datasets used and/or analysed during the current study are available from the corresponding author on reasonable request.
